# Chronic musculoskeletal pain predicted hospitalisation due to serious medical conditions in a 10 year follow up study

**DOI:** 10.1186/1471-2474-11-127

**Published:** 2010-06-18

**Authors:** Hans Lindgren, Stefan Bergman

**Affiliations:** 1 Research and Development Centre Spenshult, Spenshult Hospital for Rheumatic diseases, SE-313 92 Oskarström, Sweden; 2 Department of Radiology, Helsingborg County Hospital, SE-251 87 Helsingborg, Sweden

## Abstract

**Background:**

The aim was to examine if self reported chronic regional pain (CRP) and chronic widespread pain (CWP) predicted inpatient care due to serious medical conditions such as cerebrovascular diseases, ischemic heart diseases, neoplasms and infectious diseases in a general population cohort over a ten year follow-up period.

**Methods:**

A ten-year follow up of a cohort from the general adult population in two health care districts with mixed urban and rural population in the south of Sweden, that in 1995 participated in a survey on health and musculoskeletal pain experience. Information on hospitalisation for each subject was taken from the regional health care register. Multiple logistic regression analyses were used to study the associations between chronic musculoskeletal pain and different medical conditions as causes of hospitalisation.

**Results:**

A report of CRP (OR = 1.6; p < 0.001) or CWP ( OR = 2.1; p < 0.001) predicted at least one episode of inpatient care over a ten year period, with an increased risk in almost all diagnostic subgroups, including cerebrovascular diseases, ischemic heart diseases, and infectious diseases. There was however no increased risk of hospitalisation due to neoplasms.

**Conclusions:**

The presence of especially CWP was associated with hospital inpatient care due to several serious medical disorders. This may imply a general vulnerability to different medical conditions that has to be addressed in the assessment and management of subjects with chronic musculoskeletal pain.

## Background

Chronic musculoskeletal pain is common in the general population with a prevalence of 35-50% [1-3].This includes various painful local or regional musculoskeletal disorders, but also subjects with chronic widespread pain (CWP). CWP, with a prevalence of 11% [2-4], may reflect a musculoskeletal or other underlying organic disease, but this is reported only in a small proportion of subjects. Despite the possible lack of pathological findings, chronic musculoskeletal pain and especially CWP is known to have a great impact on self-reported health and also to be a common cause for visits in primary care [5-7]. CWP has been reported to be associated with increased cancer mortality [8], due to both a higher incidence of cancer and a reduced cancer survival [9], but this was not confirmed in a more recent report from a large population study [10].

Patients in primary care who report chronic musculoskeletal pain subsequently seek primary care for other morbidities more often than patients without pain [11]. Musculoskeletal pain and fibromyalgia are prevalent among hospitalised patients on internal medical wards [12], and physical symptoms such as musculoskeletal pain predicts hospitalisation and mortality in elderly primary care patients [13]. Little is known about the relationship between chronic pain and inpatient care for other causes than musculoskeletal pain.

The aim of this study was to examine if self reported chronic regional pain (CRP) and CWP predicted inpatient care in a general population cohort over a ten year follow-up period. Special focus was on care due to serious conditions such as cerebrovascular diseases, ischemic heart diseases, neoplasms and infectious diseases.

## Methods

### Design and subjects

A ten year longitudinal study of hospitalisation in the general population with regard to baseline musculoskeletal pain report, based on an initial postal survey and information consecutively collected in a health care register. The target population was all of the 70704 inhabitants aged 20-74 years in two healthcare districts with mixed urban and rural population on the west coast of Sweden. A representative sample of 3928 subjects was selected from the official computerised population register. The 2425 subjects that answered the baseline postal survey were considered for the study. There were 147 of these subjects that in a written consent declared that they did not want to be part of a register study. The remaining 2278 subjects in the cohort were followed from June 1995 to December 2004 or until they moved from the healthcare districts or died.

### Questionnaire

The baseline questionnaire included questions on the experience and location of chronic pain in the musculoskeletal system, other health problems, lifestyle factors, and socio-demographic background factors. There was an overall key question on chronic musculoskeletal pain: *Have you experienced pain lasting more than three months during the last twelve months? *An introduction to the question explained that the pain should be persistent or regularly recurrent *in the musculoskeletal system*. The validity of the question has been confirmed in earlier studies [2]. Pain was considered to be chronic if it had been persistent or recurrent for more than three months during the last twelve months. Location and distribution of pain was reported by a drawing of the body with 18 predefined regions [2]. A distinction was made between chronic regional pain (CRP) and chronic widespread pain (CWP) defined according to the ACR 1990 criteria for fibromyalgia [14]. Pain was considered as CWP when present for at least three months in both the left and right side of the body, and also above and below the waist. In addition axial skeletal pain (i.e. in the cervical spine, the anterior chest, the thoracic spine or the lower back) should be present. This definition of CWP has also been used in prior population studies [2-4]. When chronic pain was present but criteria for a widespread condition were not met, the subject was classified as having CRP.

*Socio-economic classification *was based on self reported main occupation at the time of the survey, and the respondents were classified according to Swedish socio-economic classification (SEI) [15]. The eighteen basic socio-economic classes were merged into four groups: Manual workers, assistant non-manual employees, intermediate/higher non-manual employees including upper-level executives, and others. The group "others" included self-employed, farmers, housewives and students. *Smoking *habit was assessed with 3 multiple-choice alternatives: 'No, never smoked regularly', 'No, have stopped', and 'Yes'. The result was dichotomised into 'Never smokers" and 'Ever smokers'.

### Healthcare register

The regional health care register consecutively collects information on all hospital care periods in the actual health care districts and in the surrounding county belonging to the same health authorities. It covers regional secondary care but not tertiary care in university hospitals. This constituted however less than 5% of the total number of hospitalisation episodes in the studied population, according to health authorities statistics in the studied area (personal communication). The register includes the date and length of the care period together with main and secondary diagnoses according to the ICD 10 classification. The research team linked information in the healthcare register to questionnaire data by means of each subject's unique Swedish social security number.

### Classification of diagnoses

Diagnoses in the health care register were, due to the long follow-up time, based on two different classifications, and were all converted into the current ICD 10 system [16]. Care due to normal child birth was excluded. Based on the main diagnose according to ICD 10 each hospital care period was attributed to one of eleven categorisation groups: (1) infectious diseases (ICD10 A00-B99; J00-J22; N10;N39), (2) malignant (ICD10 C00-C97) and benign (D00-D89) neoplasms, (3) other medical diseases (ICD10 D50-D89; E00-E90; L00-L99; G00-G44; G47-G99; H00-H95; J30-J39; J40-J99), (4) mental and behavioural disorders including drug abuse (ICD10 F00-F99), (5) cerebrovascular disease (ICD10 G45-G46; I60-I69) and ischemic heart disease (ICD10 I120-I125), (6) other diseases of the circulatory system (ICD10 I10-I14; I26-I49; I51-I52; I70-I99), (7) diseases of the digestive system (ICD10 K00-K93), (8) diseases of the musculoskeletal system (ICD10 M00-M99), (9) diseases of the genitourinary system, excluding infectious disease (ICD10 N00-N99; O00-O99), (10) symptoms and observations (ICD10 R00-R99; Z00-Z98), (11) injuries, poisoning and other external causes (ICD10 S00-Y98).

### Ethics

The study was approved by the Ethics Research Committee, Faculty of Medicine, University of Lund, Sweden, LU 843-02. Subjects that in a written consent declared that they did not want to be part of a register study were excluded. The computerised registration was approved by the Swedish Data Inspection Board.

### Statistics

Multiple logistic regression analyses were used to study the associations between chronic regional and widespread musculoskeletal pain and different causes of hospitalisation.

The statistical analyses were done with the statistical package SPSS 16.0 for Windows. Multivariable logistic regression analyses with computation of Odds Ratios (OR) were performed with simple contrast to a reference group for each of the independent variables; age, sex, baseline pain group, SEI, smoking habits, and follow-up time. Other candidate explanatory variables (marital status, emotional support, immigrant status, regular exercise, and education) were omitted from the final models because of lack of association when introduced stepwise in an analysis with regard to all causes of hospitalisation.

## Results

The 2278 included subjects had a mean age of 46.8 years and 53.2% were women.

The mean follow up time was 8.9 years. Altogether there were 2933 hospitalisation episodes and 1014 subjects (44.5%) had at least one episode of hospitalisation during follow up (Table 1). At baseline 1372 subjects (60.2%) reported no chronic pain (NCP), 588 subjects (24.5%) reported chronic regional pain (CRP), 285 subjects (12.5%) reported chronic widespread pain (CWP), and 63 subjects (2.8%) could not be classified. There were no significant differences in follow up time between the pain groups.

**Table 1:** Number and percentage of the total 2278 subjects with at least one episode of inpatient care within each of the diagnostic groups.

Diagnostic group	n =	%
Infectious disease	112	4.9
Neoplasm (malignant and benign)	144	6.3
Other medical diseases	160	7.0
Mental and behavioural disorders	47	2.1
Cerebrovascular and ischemic heart disease	144	6.3
Other diseases of the circulatory system	130	5.7
Diseases of the digestive system	168	7.4
Diseases of the musculoskeletal system	139	6.1
Diseases of the genitourinary system (excl infections)	187	8.2
Symptoms and observations	345	15.1
Injuries, poisoning and other external causes	217	9.5

The predictive value of pain group at baseline with regard to risk of hospitalisation, controlled for age, sex, socio-economics, smoking habits and follow-up time, was explored in multivariate analyses. The overall risk of being hospitalised regardless of cause was significantly increased in subjects reporting CRP, with an odds ratio (OR) of 1.6 (p < 0.001), and CWP, with an OR of 2.2 (p < 0.001), when compared to the group with NCP (Table 2).

**Table 2:** Odds Ratios (OR) for the associations between pain groups at baseline and hospitalisation, controlled for age, sex, socio-economic, smoking habits and follow-up time.

		All causes		Infectious diseases		Neoplasms		Other medical diseases	
	No	OR (95% CI)	p-value	OR (95% CI)	p-value	OR (95% CI)	p-value	OR (95% CI)	p-value
Sex									
Men	1065	1		1		1		1	
Women	1213	1.0 (0.8-1.2)	0.996	0.8 (0.5-1.1)	0.162	1.4 (1.0-2.0)	0.078	1.0 (0.7-1.4)	0.968
Age (years)									
20-33	559	1		1		1		1	
34-46	541	1.0 (0.7-1.3)	0.816	0.3 (0.1-0.7)	0.006	8.7 (2.6-29.7)	0.001	0.6 (0.3-1.1)	0.080
47-58	583	1.4 (1.1-1.8)	0.016	1.1 (0.6-2.1)	0.676	15.3 (4.6-50.1)	<0.001	0.8 (0.5-1.3)	0.419
59-74	595	3.2 (2.5-4.1)	<0.001	2.4 (1.4-4.1)	0.002	28.4 (8.8-91.5)	<0.001	1.7 (1.1-2.6)	0.016
Pain group^a^									
NCP	1372	1		1		1		1	
CRP	558	1.6 (1.3-1.9)	<0.001	1,6 (1.0-2.5)	0.044	1.1 (0.8-1.7)	0.528	1.3 (0.8-1.9)	0.270
CWP	285	2.2 (1.7-2.9)	<0.001	2,0 (1.2-3.5)	0.011	1.1 (0.6-1.8)	0.773	2.0 (1.3-3.1)	0.003
Socioeconomic group^b^									
Group A	556	1		1		1		1	
Group B	309	1.3 (1.0-1.8)	0.056	1.0 (0.5-1.9)	0.978	1.3 (0.7-2.3)	0.393	1.0 (0.6-1.9)	0.975
Group C	1103	1.2 (1.0-1.5)	0.062	0.8 (0.5-1.3)	0.317	1.1 (0.7-1.7)	0.767	1.2 (0.8-1.9)	0.344
Others	310	1.4 (1.0-1.9)	0.037	0.8 (0.4-1.6)	0.608	1.0 (0.5-1.8)	0.891	1.4 (0.8-2.5)	0.241
Smoking habit^c^									
Never	1163	1		1		1		1	
Ever	1106	1.3 (1.1-1.5)	0.008	1.4 (0.9-2.1)	0.090	2.3 (1.6-3.5)	<0.001	1.5 (1.1-2.1)	0.018
Follow up time	2278	1.1 (1.0-1.1)	0.001	1.0 (0.9-1.1)	0.402	0.9 (0.8-0.9)	<0.001	1.0 (0.9-1.0)	0.193

^a ^Data not shown for the group of subjects (n = 63) that could not be classified

^b ^Group A: Intermediate/higher non-manual employees and upper-level executives; Group B: Assistant non-manual employees;

Group C: Manual workers

^c ^Data not shown for subjects with unknown smoking habit (n = 9)

Separate analyses based on the main diagnosis according to ICD 10 were done in eleven diagnostic subgroups (Table 2, 3, 4). The risk of inpatient hospital care due to infectious diseases was significantly increased both in subjects reporting CRP (OR 1.6; p = 0.044) and CWP (OR 2.0; p = 0.011). There was no increased risk of care for neoplasms in any of the two pain groups; CRP (OR 1.1; p = 0.528) and CWP (OR 1.1; P = 0.773). A separate analysis (not shown in table) was done for the subgroup with known malignant disease (n = 111) with the same result in subjects with CRP (OR 1.2; p = 0.432) and CWP (OR 1.1; p = 0.817).

**Table 3:** Odds Ratios (OR) for the associations between pain groups at baseline and hospitalisation, controlled for age, sex, socio-economic, smoking habits and follow-up time.

		Mental disorders		Cerebrovascular and ischemic heart disease		Other diseases of the circulatory system		Diseases of the digestive system	
	No	OR (95% CI)	p-value	OR (95% CI)	p-value	OR (95% CI)	p-value	OR (95% CI)	p-value
Sex									
Men	1065	1		1		1		1	
Women	1213	1.8 (1.0-3.4)	0.068	0.5 (0.4-0.8)	0.001	0.5 (0.3-0.7)	<0.001	0.9 (0.6-1.2)	0.423
Age (years)									
20-33	559	1		1		1		1	
34-46	541	0.8 (0.3-2.0)	0.622	8.7 (2.0-38.6)	0.004	1.7 (0.6-5.0)	0.309	1.3 (0.7-2.4)	0.325
47-58	583	1.1 (0.5-2.6)	0.822	18.6 (4.4-78.8)	<0.001	6.7 (2.8-16.6)	<0.001	1.9 (1.1-3.2)	0.028
59-74	595	0.9 (0.4-2.1)	0.806	48.2 (11.7-198.1)	<0.001	13.1 (5.6-30.7)	<0.001	2.8 (1.7-4.6)	<0.001
Pain group^a^									
NCP	1372	1		1		1		1	
CRP	558	0.6 (0.2-1.5)	0.267	1.6 (1.0-2.4)	0.030	1.4 (0.9-2.2)	0.139	1.4 (0.9-2.1)	0.092
CWP	285	2.5 (1.2-5.1)	0.014	1.9 (1.2-3.1)	0.006	1.8 (1.1-3.0)	0.020	2.7 (1.8-4.1)	<0.001
Socioeconomic group^b^									
Group A	556	1		1		1		1	
Group B	309	2.1 (0.6-6.7)	0.222	2.2 (1.1-4.3)	0.023	1.7 (0.8-3.4)	0.143	1.7 (0.9-2.9)	0.085
Group C	1103	2.3 (0.9-6.0)	0.102	2.4 (1.4-4.2)	0.002	2.0 (1.2-3.5)	0.013	1.6 (1.0-2.6)	0.034
Others	310	3.0 (1.0-9.2)	0.061	2.9 (1.5-5.6)	0.002	2.3 (1.2-4.5)	0.016	1.7 (0.9-3.0)	0.101
Smoking habit^c^									
Never	1163	1		1		1		1	
Ever	1106	1.4 (0.8-2.6)	0.253	1.2 (0.9-1.8)	0.272	1.2 (0.8-1.8)	0.328	1.5 (1.1-2.1)	0.022
Follow up time	2278	1.0 (0.8-1.1)	0.455	1.0 (0.9-1.1)	0.510	1.0 (0.9-1.0)	0.278	1.1(1.0-1.2)	0.231

^a ^Data not shown for the group of subjects (n = 63) that could not be classified

^b ^Group A: Intermediate/higher non-manual employees and upper-level executives; Group B: Assistant non-manual employees;

Group C: Manual workers

^c ^Data not shown for subjects with unknown smoking habit (n = 9)

**Table 4:** Odds Ratios (OR) for the associations between pain groups at baseline and hospitalisation, controlled for age, sex, socio-economic, smoking habits and follow-up time.

		Diseases of the musculoskeletal system		Diseases of the genitourinary system		Symptoms and observations		Injuries, poisoning and other external causes	
	No	OR (95%CI)	*p-value*	OR (95% CI)	p-value	OR (95% CI)	p-value	OR (95% CI)	p-value
Sex									
Men	1065	1		1		1		1	
Women	1213	0.7 (0.5-1.1)	0.090	3.7 (2.6-5.5)	<0.001	0.8 (0.6-1.0)	0.022	0.7 (0.5-1.0)	0.040
Age (years)									
20-33	559	1		1		1		1	
34-46	541	2.0 (1.0-4.0)	0.050	0.3 (0.2-0.4)	<0.001	1.3 (0.8-2.0)	0.259	1.4 (0.9-2.3)	0.173
47-58	583	2.5 (1.3-4.8)	0.006	0.3 (0.2-0.5)	<0.001	1.8 (1.2-2.8)	0.003	1.5 (0.9-2.5)	0.081
59-74	595	3.6 (1.9-6.8)	<0.001	0.4 (0.2-0.6)	<0.001	4.0 (2.7-5.8)	<0.001	3.2 (2.0-4.9)	<0.001
Pain group^a^									
NCP	1372	1		1		1		1	
CRP	558	2.5 (1.6-3.9)	<0.001	1.4 (1.0-2.1)	0.072	1.7 (1.3-2.3)	<0.001	1.4 (1.0-2.0)	0.053
CWP	285	4.1 (2.5-6.5)	<0.001	2.4 (1.5-3.6)	<0.001	2.1 (1.5-2.9)	<0.001	1.8 (1.2-2.7)	0.004
Socioeconomic group^b^									
Group A	556	1		1		1		1	
Group B	309	1.2 (0.6-2.2)	0.584	1.0 (0.6-1.6)	0.906	1.2 (0.8-1.8)	0.432	1.4 (0.8-2.2)	0.221
Group C	1103	1.2 (0.7-1.9)	0.453	0.9 (0.6-1.4)	0.666	1.4 (1.0-1.9)	0.055	1.3 (0.9-1.9)	0.209
Others	310	1.3 (0.7-2.4)	0.476	0.9 (0.5-1.6)	0.812	1.8 (1.2-2.7)	0.006	1.5 (0.9-2.5)	0.090
Smoking habit^c^									
Never	1163	1		1		1		1	
Ever	1106	0.8 (0.6-1.2)	0.275	0.9 (0.6-1.2)	0.368	1.2 (0.9-1.5)	0.262	0.9 (0.7-1.2)	0.465
Follow up time	2278	1.1(1.0-1.2)	0.182	1.2 (1.1-1.4)	0.001	1.1 (1.1-1.2)	0.001	1.1 (1.0-1.3)	0.010

^a ^Data not shown for the group of subjects (n = 63) that could not be classified

^b ^Group A: Intermediate/higher non-manual employees and upper-level executives; Group B: Assistant non-manual employees;

Group C: Manual workers

^c ^Data not shown for subjects with unknown smoking habit (n = 9)

The risk of care due to 'other medical disorders' was not increased for subjects with CPR (OR 1.3; p = 0.270) but significantly increased for those with CWP (OR 2.0; p = 0.003).

The risk of care due to psychiatric disease and drug abuse was not increased for subjects with CRP (OR 0.6; p = 0.267), but significantly increased for subjects with CWP (OR 2.5; p = 0.014).

The risk of care due to cerebrovascular and ischemic heart disease (IHD) was significantly increased for both subjects with CRP (OR 1.6; p = 0.030) and subjects with CWP (OR 1.9; p = 0.006). There was no significantly increased risk of care due to other heart diseases than IHD for subjects with CRP (OR: 1,4; p = 0.139), but for subjects with CWP the risk was significantly increased (OR:1.8; p = 0.020).

The risk of care due to disease of the digestive system was not significantly increased for subjects with CRP (OR 1.4; p = 0.092), but significantly increased for subjects with CWP (OR 2.7; p < 0.001). The risk of hospitalisation due to musculoskeletal disease was increased both for subjects with CRP (OR 2.5; p < 0.001) and CWP (OR 4.1; p < 0.001). The risk of care due to diseases of the genitourinary system (infections excluded) was not significantly increased for CRP (OR 1.4; p = 0.072), but significantly increased for CWP (OR 2.4; p < 0.001). The risk of care for symptoms and observations was significantly increased both for CRP (OR 1.7; p < 0.001) and CWP (OR 2.1; p < 0.001), and the risk of care due to injuries and poisoning was significantly increased for CWP (OR 1.7; p = 0.007), but not for CRP (OR 1.4; p = 0.053).

Since univariate analyses (not shown in tables) revealed the possibility of sex and age differences in the association between chronic pain and the risk of hospitalisation, regression analyses were also done with stratification on sex and age, controlling for all other variables. There were no sex differences in the associations between CRP or CWP and the overall risk of hospitalisation, but sex differences could be seen with regard to the different causes of hospitalisation (Table 5, 6, 7). Hospitalisation due to infections was significantly associated with CWP for women (OR 2.9; p = 0.004), but not for men (OR 1.3; p = 0.541). Care due to cerebrovascular and ischemic heart disease was significantly associated with CRP (OR 2.1; p = 0.007) and CWP (OR 2.3; p = 0.017) for men, but not for women (OR 1.0; p = 0.929, and OR 1.6; p = 0.172).

**Table 5:** Odds Ratios (OR) for the associations between pain groups at baseline and hospitalisation, stratified on sex and age, and controlled for all variables in Table 2.

			All causes		Infectious diseases		Neoplasms		Other medical diseases	
		No	OR (95% CI)	p-value	OR (95% CI)	p-value	OR (95% CI)	p-value	OR (95% CI)	p-value
Stratified on sex										
Men	NCP	687	1		1		1		1	
	CRP	258	1.5 (1.1-2.1)	0.006	1.6 (0.9-3.0)	0.115	1.4 (0.7-2.5)	0.317	1.2 (0.7-2.2)	0.442
	CWP	87	2.2 (1.4-3.7)	0.002	1.3 (0.5-3.2)	0.541	0.8 (0.3-2.0)	0.586	1.2 (0.5-2.8)	0.662
Women	NCP	685	1		1		1		1	
	CRP	300	1.5 (1.1-2.0)	0.004	1.6 (0.8-3.2)	0.217	1.0 (0.6-1.8)	0.941	1.2 (0.7-2.2)	0.486
	CWP	198	2.3 (1.6-3.2)	<0.001	2.9 (1.4-5.9)	0.004	1.2 (0.6-2.2)	0.587	2.6 (1.5-4.5)	0.001
Stratified on age										
20-33	NCP	404	1		1		1		1	
	CRP	108	2.1 (1.3-3.4)	0.002	2.0 (0.7-5.8)	0.211	1.4 (0.1-17.5)	0.783	0.8 (0.3-2.0)	0.637
	CWP	29	3.1 (1.4-7.1)	0.007	2.6 (0.5-13.4)	0.249	-	-	0.8 (0.2-3.8)	0.818
34-46	NCP	341	1		1		1		1	
	CRP	129	1.3 (0.9-2.1)	0.180	0.6 (0.1-5.1)	0.618	0.7 (0.2-2.2)	0.531	0.7 (0.2-2.3)	0.577
	CWP	54	1.6 (0.8-2.9)	0.158	-	-	1.0 (0.3-3.7)	0.963	1.6 (0.4-6.2)	0.494
47-58	NCP	324	1		1		1		1	
	CRP	159	2.0 (1.4-3.0)	0.001	2.3 (1.0-5.3)	0.056	1.8 (0.9-3.8)	0.115	3.0 (1.2-7.2)	0.015
	CWP	92	3.1 (1.9-5.2)	<0.001	1.2 (0.4-4.3)	0.728	1.3 (0.5-3.3)	0.568	3.3 (1.2-9.0)	0.021
59-74	NCP	303	1		1		1		1	
	CRP	162	1.1 (0.7-1.6)	0.806	1.3 (0.6-2.5)	0.479	1.0 (0.6-1.8)	0.995	1.1 (0.6-2.0)	0.841
	CWP	110	1.9 (1.1-3.1)	0.013	2.5 (1.2-5.0)	0.010	1.0 (0.5-2.1)	0.938	2-3 (1.2-4.5)	0.011

**Table 6:** Odds Ratios (OR) for the associations between pain groups at baseline and hospitalisation, stratified on sex and age, and controlled for all variables in Table 2.

			Mental disorders		Cerebrovascular and ischemic heart disease		Other diseases of the circulatory system			Diseases of the digestive system	
		No	OR (95% CI)	p-value	OR (95% CI)	p-value	OR (95% CI)	OR (95% CI)	p-value		OR (95% CI)
Stratified on sex											
Men	NCP	687	1		1		1			1	
	CRP	258	0.9 (0.2-3.8)	0.935	2.1 (1.2-3.5)	0.007	1.5 (0.9-2.6)	0.146		1.2 (0.7-2.1)	0.506
	CWP	87	3.1 (0.7-13.1)	0.125	2.3 (1.2-4.6)	0.017	1.6 (0.8-3.4)	0.186		1.9 (1.0-3.8)	0.060
Women	NCP	685	1		1		1			1	
	CRP	300	0.4 (0.1-1.5)	0.169	1.0 (0.5-2.1)	0.929	1.2 (0.6-2.5)	0.646		1.7 (0.9-2.9)	0.076
	CWP	198	2.2 (1.0-5.1)	0.056	1.6 (0.8-3.1)	0.172	1.9 (0.9-3.8)	0.087		3.5 (2.0-6.0)	<0.001
Stratified on age											
20-33	NCP	404	1		1		1			1	
	CRP	108	1.3 (0.2-6.6)	0.774	3.3 (0.2-55.0)	0.401	-	-		1.3 (0.5-3.7)	0.623
	CWP	29	7.3 (1.6-33.4)	0.011	-	-	-	-		0.6 (0.1-5.3)	0.676
34-46	NCP	341	1		1		1			1	
	CRP	129	1.3 (0.2-7.1)	0.799	0.9 (0.2-3.5)	0.843	0.9 (0.2-4.9)	0.926		1.6 (0.7-4.0)	0.263
	CWP	54	1.2 (0.1-11.5)	0.882	5.4 (1.3-22.4)	0.021	3.0 (0.5-17.7)	0.220		3.4 (1.2-9.3)	0.021
47-58	NCP	324	1		1		1			1	
	CRP	159	0.3 (0.0-2.6)	0.286	1.9 (0.9-4.2)	0.115	2.7 (1.2-5.9)	0.013		1.0 (0.5-2.1)	0.988
	CWP	92	2.5 (0.7-9.2)	0.160	1.9 (0.7-4.9)	0.183	2.7 (1.0-7.2)	0.044		2.2 (1.0-4.7)	0.047
59-74	NCP	303	1		1		1			1	
	CRP	162	0.2 (0.0-1.7)	0.141	1.6 (0.9-2.7)	0.096	1.2 (0.6-2.1)	0.585		1.6 (0.8-3.0)	0.150
	CWP	110	1.4 (0.4-4.9)	0.559	1.8 (1.0-3.2)	0.068	1.7 (0.9-3.2)	0.124		3.5 (1.9-6.6)	<0.001

**Table 7:** Odds Ratios (OR) for the associations between pain groups at baseline and hospitalisation, stratified on sex and age, and controlled for all variables in Table 2.

			Diseases of the musculoskeletal system		Diseases of the genitourinary system		Symptoms and observations		Injuries, poisoning and other external causes	
		No	OR (95% CI)	p-value	OR (95% CI)	p-value	OR (95% CI)	p-value	OR (95% CI)	p-value
Stratified on sex										
Men	NCP	687	1		1		1		1	
	CRP	258	2.2 (1.2-3.9)	0.009	1.4 (0.6-3.3)	0.453	1.9 (1.3-2.8)	0.001	1.5 (1.0-2.4)	0.085
	CWP	87	3.2 (1.5-6.7)	0.002	5.1 (2.2-11.9)	<0.001	1.7 (1.0-2.9)	0.073	1.9 (1.0-3.6)	0.057
Women	NCP	685	1		1		1		1	
	CRP	300	2.9 (1.5-5.4)	0.001	1.5 (1.0-2.3)	0.066	1.5 (1.0-2.3)	0.042	1.3 (0.8-2.1)	0.376
	CWP	198	4.8 (2.5-9.2)	<0.001	2.0 (1.2-3.4)	0.007	2.4 (1.6-3.7)	<0.001	1.8 (1.1-3.0)	0.032
Stratified on age										
20-33	NCP	404	1		1		1		1	
	CRP	108	5.8 (1.5-22.1)	0.010	1.6 (0.8-3.1)	0.158	2.2 (1.1-4.6)	0.037	1.9 (0.8-4.5)	0.131
	CWP	29	12.5 (2.4-65.8)	0.003	6.7 (2.4-18.4)	<0.001	3.0 (1.0-9.0)	0.052	0.8 (0.1-6.4)	0.840
34-46	NCP	341	1		1		1		1	
	CRP	129	2.0 (0.8-4.8)	0.142	1.5 (0.6-3.8)	0.447	1.4 (0.7-2.8)	0.284	1.6 (0.8-3.2)	0.220
	CWP	54	2.0 (0.6-6.6)	0.272	2.0 (0.7-5.7)	0.194	1.4 (0.5-3.9)	0.537	1.1 (0.3-3.9)	0.928
47-58	NCP	324	1		1		1		1	
	CRP	159	7.6 (2.9-19.5)	<0.001	2.0 (0.9-4.6)	0.080	1.4 (0.8-2.4)	0.240	1.3 (0.7-2.6)	0.434
	CWP	92	10.8 (3.8-30.3)	<0.001	3.2 (1.3-7.8)	0.009	2.1 (1.2-4.0)	0.016	1.2 (0.5-2.8)	0.646
59-74	NCP	303	1		1		1		1	
	CRP	162	1.2 (0.6-2.4)	0.697	0.8 (0.4-1.8)	0.641	1.9 (1.2-3.0)	0.003	1.2 (0.7-2.1)	0.527
	CWP	110	2.7 (1.4-5.3)	0.004	1.4 (0.6-2.9)	0.447	2.1 (1.3-3.5)	0.002	2.5 (1.4-4.2)	0.001

Stratification on age revealed that there was an association between CWP and all causes of hospitalisation in the youngest and the two oldest age groups, but not in the second youngest, aged 34-46. This pattern was relatively consistent also when split to the different causes of care. One exception was care due to cerebrovascular and ischemic heart disease that was strongly (OR 5.4; p = 0.021) associated with CWP in this second age group.

## Discussion

A baseline report of CRP or CWP predicted at least one episode of inpatient care due to serious medical disorders over a ten year period, when compared to subjects without chronic pain. Adjusted for sex, age, smoking, socio-economic background, and follow up time this was true for almost all diagnostic subgroups, and the associations were more frequent and stronger for CWP than for CRP. There were also some notable differences between men and women, where CWP was associated with care due to infections in women, but not in men, and associated with care due to cerebrovascular and ischemic heart disease in men, but not in women.

Previous studies have proposed that hospitalisation of elderly primary care patients could be predicted by musculoskeletal pain [13]. The present study supports that this is true also from a general population perspective including ages 20-74. Analyses stratified on age highlighted that also younger subjects with CWP had an increased risk of hospitalisation. The association between especially CWP and the risk of hospital care is also supported by previous findings that CWP and fibromyalgia are frequent among patients on internal medical wards [12], but also raises the question whether some of frequently reported symptoms such as fatigue in hospitalised patients could be part of a undiagnosed pain syndrome. It has for example been suggested that bodily pain has a high impact on health status in patients hospitalised with heart failure [17].

Somatisation has been shown to be associated with the development of CWP [18], and CWP has been reported to precede other medical conditions in primary care [11]. The findings in this study emphasise that CWP also is associated with serious medical conditions needing hospital care, such as infectious diseases, ischemic heart disease and cerebrovascular diseases. There was however no increased risk of hospitalisation due to neoplasms.

Both subjects with CRP and CWP had an increased risk of hospital care due to ischemic heart disease and cerebrovascular disease, with a higher risk in those with CWP. When stratifying the analysis on sex it was seen that the association between CRP or CWP and sex mainly was an effect of such an association for men but not for women. The association between CWP and cardiovascular disease is somewhat supported by the report of a nearly significant association between CWP and an increased mortality in cardiovascular disease in a recent study [19]. The association is not explained by smoking habits and the mechanism remains unclear.

There was no association between CRP or CWP and hospital care due to neoplasms. A previous study reported that subjects with CWP were twice as likely to die from cancer over a nine year period as subjects with no pain [9], and this result was also supported by a more recent study from the same research group [19]. This relationship between self reported widespread pain and cancer death was on the other hand not confirmed in a study from a large rural population in Finland [10], or in a recent study from UK [20]. There are a couple of plausible explanations to the different results. It could be that there is no real link between chronic musculoskeletal pain and incidence of cancer, supported by the fact that no clear biological mechanism is known [9]. There could also be differences in lifestyle and personal factors in the different studied populations. In the present study it was noted that the association between CWP and hospitalisation due to neoplasms was confounded by smoking, which more than doubled the risk of inpatient care due to cancer. The results in previous studies have not been full controlled for smoking habits. It must also be pointed out that the present study deals with inpatient hospital care for subjects with different pain group belongings, and not with the incidence of and survival in cancer. Hospital care might not be the optimal measure of the relationship between chronic pain and cancer and further analysis of data from the national cancer register and death register might give additional important information.

The increased risk of hospitalisation due to other causes than musculoskeletal disorders highlights that chronic musculoskeletal pain could be an expression of a more general vulnerability to disease and health problems. The variation with different causes of care for men and women also suggests that the association is part of a multifactorial process. This study could not ascertain that the subjects were free of the medical disorders at the start, and the results thus suggest an association and not necessarily a causal relationship between chronic musculoskeletal pain and the medical conditions. This also means that the chronic pain at baseline (CRP or CWP) might have been an expression or symptom of pre-existing disease, such as cardiovascular, which subsequently led to hospitalisation. An interesting finding in the present study is that there is a difference between CRP and CWP in the risk of hospitalisation, where a report of CWP more frequently and stronger predicted hospitalisation compared to a report of NCP or CRP, notably also for those in the youngest age group. This could indicate that subjects with CWP are more vulnerable to disease than subjects with CRP or NCP. One explanation could be that distress is a common factor for both development of chronic pain (especially CWP) and various medical disorders. It has recently been shown that psychosocial distress has a strong aetiological influence on CWP while this link is weaker with CRP [21]. Observational studies have also linked psychosocial factors to cardiovascular disease [22].

The strength of this paper is that it is based on a cohort from the general population that has been followed prospectively over time with the help of official health care registers. The frequency of inpatient care for some less common serious disorders could have been marginally underestimated due to that tertiary care in university hospital not was included. This constituted however less than 5% of the total number of hospitalisation episodes in the studied population and was not supposed to change the overall result. Age, sex, socio-economic status, smoking habits, and follow up time were likely to be confounders, and were controlled for in the analyses. A selection bias due to non-response in the first cross-sectional survey, where subjects with chronic pain were more prone to respond than people without chronic pain is assumed not to affect the conclusions in this longitudinal study [2]. Another possible problem could be errors or misclassification of subjects with respect to pain sub-groups, socio-economic factors, and hospital diagnoses. The material was thoroughly checked for errors. Misclassifications of exposures or outcomes were likely to be non-differential between subjects without pain or subjects having CRP or CWP.

## Conclusions

The presence of chronic musculoskeletal pain, especially CWP, was associated with hospitalisation due to several serious medical disorders including cerebrovascular disease, ischemic heart disease, and infections, but not neoplams. This may imply a general vulnerability to different medical conditions that has to be addressed in the assessment and management of subjects with chronic musculoskeletal pain.

## Competing interests

The authors declare that they have no competing interests.

## Authors' contributions

SB carried out the baseline studies that provided questionnaire data. Both HB and SB designed the study, carried out statistical analyses and drafted the manuscript. Both authors approved the final manuscript.

## Pre-publication history

The pre-publication history for this paper can be accessed here:

http://www.biomedcentral.com/1471-2474/11/127/prepub
